# Real-Time Effects of Payer Restrictions on Reproductive Healthcare: A Qualitative Analysis of Cost-Related Barriers and Their Consequences among U.S. Abortion Seekers on Reddit

**DOI:** 10.3390/ijerph18179013

**Published:** 2021-08-26

**Authors:** Jenny A. Higgins, Madison Lands, Taryn M. Valley, Emma Carpenter, Laura Jacques

**Affiliations:** 1 Department of Obstetrics and Gynecology, University of Wisconsin-Madison, Madison, WI 53715, USA; lands@wisc.edu (M.L.); tmvalley@wisc.edu (T.M.V.); emma.carpenter@utexas.edu (E.C.); laura.jacques@wisc.edu (L.J.); 2 Collaborative for Reproductive Equity (CORE), University of Wisconsin-Madison, Medical Sciences Center 4245, Madison, WI 53706, USA; 3 Department of Anthropology, University of Wisconsin-Madison, Madison, WI 53706, USA; 4 Presidential Management Fellows Program, U.S. Office of Personnel Management, Washington, DC 20415, USA

**Keywords:** abortion seekers, abortion clinics, abortion (induced), abortion (illegal), Medicaid, third-party payers, social media, reproductive health services, reproductive rights

## Abstract

Objective: The Hyde Amendment and related policies limit or prohibit Medicaid coverage of abortion services in the United States. Most research on cost-related abortion barriers relies on clinic-based samples, but people who desire abortions may never make it to a healthcare center. To examine a novel, pre-abortion population, we analyzed a unique qualitative dataset of posts from Reddit, a widely used social media platform increasingly leveraged by researchers, to assess financial obstacles among anonymous posters considering abortion. Methods: In February 2020, we used Python to web-scrape the 250 most recent posts that mentioned abortion, removing all identifying information and usernames. After transferring all posts into NVivo, a qualitative software package, the team identified all datapoints related to cost. Three qualitatively trained evaluators established and applied codes, reaching saturation after 194 posts. The research team used a descriptive qualitative approach, using both inductive and deductive elements, to identify and analyze themes related to financial barriers. Results: We documented multiple cost-related deterrents, including lack of funds for both the procedure and attendant travel costs, inability to afford desired abortion modality (i.e., medication or surgical), and for some, consideration of self-managed abortion options due to cost barriers. Conclusions: Findings from this study underscore the centrality of cost barriers and third-party payer restrictions to stymying reproductive health access in the United States. Results may contribute to the growing evidence base and building political momentum focused on repealing the Hyde Amendment.

## 1. Introduction

Although a frequently sought healthcare procedure, abortion can be very challenging to obtain in the U.S. Barriers include a sociopolitical landscape of abortion restrictions and clinic closures, deep-set cultural stigma, and difficulty traveling to the nearest abortion clinic [1]. However, one of the most powerful obstacles to abortion care is cost [2,3].

Three years after 1973′s Roe v. Wade decision, Illinois Representative Henry Hyde and colleagues attached a cost-related provision to the appropriations bill for the Departments of Labor, Health, Education, and Welfare (now the Department of Health and Human Services). Widely known as “the Hyde Amendment”, this provision ended federal funding, including Medicaid funding, for abortion. In the years between Roe’s passage and the implementation of the Hyde Amendment, approximately one in three abortions were paid for by Medicaid [4]. This large proportion of clients using the public safety net to pay for abortion services is consistent with literature documenting that poverty is strongly associated with unwanted pregnancies [5].

Seventeen states now allow state-level Medicaid dollars to cover abortion services [6]. However, most reproductive-age people in the U.S. live in places where publicly subsidized healthcare covers prenatal and birthing care but not pregnancy termination. Even where coverage is available, challenges in navigating the Medicaid system can lead to delays in abortion care or inability to receive care altogether [4]. In one study, individuals in states that restrict Medicaid funding for abortion were more likely than their counterparts in Medicaid-covered states to still be seeking abortion services one month after study baseline [7]. Recent legislation has imposed increased abortion-related restrictions on private insurance as well.

While costs vary, the typical price tag for both surgical abortion and medication abortion in the first trimester usually falls between $500 and $1000 [8,9], with the average medication abortion price hovering around $550 [10]. Second trimester costs are significantly higher [9]. However, those procedure fees do not include attendant transportation, childcare, or other costs such as loss of wages or potential job loss from taking time off from work. Such concomitant costs may be especially significant for those living far away from a clinic—a population that has swelled as abortion healthcare facilities have closed nationwide [11]. Many states require 24–72-h waiting periods and unnecessary multiple clinic visits, which also compound abortion-related costs [12]. In 2019, 37% of Americans surveyed would not be able to cover a $400 unexpected expense on their own [13]. In the landmark Turnaway Study, the out-of-pocket expenses constituted one-third of monthly income for over one-third of abortion seekers [14].

Given lack of insurance coverage, an estimated 70–75% of patients pay out of pocket for abortion services [2,15]. Some financial assistance exists, either by way of grassroots abortion funds or philanthropic grants [8]. Moreover, financial assistance rarely covers all abortion-related costs, meaning that patients must still raise sizable capital.

### Limitations of Clinic-Based Research Regarding Abortion Cost Barriers

Most research on cost-related barriers focuses on clinic-based populations—people who visited an abortion clinic. However, many who desire abortion services never get through the doors of a healthcare center, as extensive legal and attendant logistical barriers make in-person clinic access difficult. A large body of research documents that people seeking abortion face both formal and informal barriers, including lack of clarity about how to actually obtain services [16], significant travel distances [17], medically unnecessary clinical standards [18] and regulation that can contribute to clinic closures, and gestational limitations [19]. While it is difficult to quantify the full impact of these barriers, this cumulative evidence suggests that people who at least consider abortions never get to a clinic for desired services [16]. For example, in counties in Wisconsin that lost the greatest access to abortion services after clinic closures, researchers observed reduced abortion rates and increased birth rates, suggesting that at least some people who would have wished to access abortions were unable to do so [20]. In one study of people presenting for prenatal care at healthcare facilities, researchers found that approximately 30% had at least briefly considered an abortion, but only 2–3% had visited an abortion clinic [21]. Documenting real-time versus retrospective reports of obstacles before people get to a clinic, if ever, is a critical contribution to the evidence base regarding the burdens of payer cost restrictions.

Quantitative surveys and other close-ended research methods can generate highly valuable data about the prevalence of cost barriers. However, they are not equipped to communicate the ways in which abortion seekers deal with healthcare costs nor the urgency or intensity of these cost barriers. Qualitative data collection can illuminate more elaborate domains of people’s pre-abortion experiences, highlighting the ways in which health policies manifest in people’s lives. Moreover, qualitative analysis of existing (versus solicited) data can highlight what matters most to individuals themselves without inserting pre-formulated research questions or concepts.

Qualitative analyses of anonymous social media fora hold promise for such a study. The overwhelming majority of U.S. adults access health-related information online [22], including information about abortion [23]. Reddit is a website of user-aggregated content dispersed over two million user-created and monitored message boards. Approximately one-quarter of the U.S. young adults use Reddit [24], and in December 2020, just prior to data collection for this study, Reddit was the seventh most visited website in the country [25]. Given the benefits of copious, anonymous, real-time accounts of people seeking abortions, we set out to document cost-related barriers to obtaining desired abortions by analyzing a novel dataset of Reddit posts.

## 2. Materials and Methods

### 2.1. Reddit as an Emerging Qualitative Data Source

Reddit-based data collection has a number of advantages, including its widespread use, gratis nature, and abundant personal narratives that researchers may miss with more traditional data collection modes such as surveys or interviews [26]. The anonymous nature of Reddit makes it especially well-suited for studies of socially stigmatized experiences [27] such as abortion. Researchers have used Reddit data to examine a variety of health-related topics, including vaccines for human papillomavirus and prenatal testing [26,28,29].

### 2.2. Data Collection

Using Python, researchers web-scraped 250 posts from reproductive-health-related sub-Reddits, which are topic-based message boards. Investigators scraped these posts on 12 February 2020 with posts occurring between 29 January and 12 February—just before COVID-19-related lockdowns. We selected 250 as an *N* based on prior Reddit research [27] and later reached theoretical saturation within the first 200 (*n* = 194).

During the scraping and database-creation processes, investigators removed all identifying information, including posters’ usernames. The study team excluded posts from outside the U.S. We transferred the posts to NVivo (QSR International, Melbourne, Austrailia,2019), which is a qualitative analysis software package.

Since all posts were anonymous, not linked to IP addresses, and in the public domain, we did not (and could not) seek explicit consent from Reddit posters. This study was approved by the University of Wisconsin-Madison’s Health Sciences Institutional Review Board (Ethical Approval Code: 2019-0697).

### 2.3. Analysis

We employed a qualitative descriptive approach in that we used data to describe posters’ experiences versus using data to build theory or imbue descriptions with theoretical meaning [30]. We also used a combined inductive and deductive approach: we analyzed data based on our original research questions and hypotheses as well as codes and themes that arose from the data themselves [31].

Our qualitative team represented two PhD-level researchers with significantly qualitative research expertise and two masters-level researchers with qualitative training and prior experience. (The final team member and fifth author reviewed final memos and contributed to theme and manuscript development.) These four study team members independently read a random subset of posts, prepared lists of emerging themes, and met to discuss and refine these themes into potential codes. These codes augmented the list of pre-established concepts identified by the investigators’ research questions and the existing literature. Once reaching thematic agreement, we created a codebook with code names, definitions, information on when to apply, and illustrative quotations. A few additional themes emerged during the coding process, and investigators circled back to recode prior posts accordingly. The final codebook contained 26 codes.

Three members of the study team systematically coded each post within NVivo 12. They first independently coded 25 posts; then, they met to resolve discrepancies and refine consistency in code application. Once they achieved interrater reliability of greater than 80%, measured using an Alpha score in NVivo, team members singly coded the remaining posts. Coders met weekly to discuss questions or confirm codes. They stopped coding after 194 posts, as they had reached theoretical saturation.

We analyzed all cost-related coding reports, which are documents containing every single appearance of a code in the dataset. Coding reports included “logistical aspects of abortion experience,” “more general barriers to abortion,” and “factors influencing method choice.” We also searched the larger dataset for posts using any of the following terms: *money, finances, expensive, cost, afford, $, dollar(s), and insurance.*

Then, the first and second author independently read all the above reports, made notes on themes, and met to discuss. The second author systematically reviewed the reports and identified all quotations supporting each of the main concepts. The first author wrote up memos with preliminary descriptions and illustrative quotations for each theme; then, they shared with all other authors to review and refine. The final vetted four themes appear below.

## 3. Results

Approximately one-fourth (*n* = 47) of the 194 posts included in this analysis mentioned some sort of financial constraint in people’s abortion-seeking processes. Analyses revealed four coherent and well-statured themes related to abortion cost.

### 3.1. Inability to Secure Funds for Abortion Services and Transportation

Most commonly, posters described inability to secure the funds needed to obtain the abortion. The following quote illustrates barriers to procedure costs themselves:

*(The clinic) said it would be $550. I haven’t been able to come up with that due to bills and finding out so late. I’ve been applying for loans but my credit score is 500. By my appointment time I will be 8 weeks along. What have you done when you need money quick?*

Another example highlighted the emotional costs of such funding challenges:

*(The clinic) was no help. I was essentially turned away because I hadn’t heard back from the funding assistance places they’d told me about, and I was too broke to be able to afford any of the procedures. I felt stuck, powerless, and defeated.*

Reddit users also struggled to afford the transportation costs necessary to obtain abortions—particularly for those living significant distances from the closest provider. These costs exacerbated the financial burden of the procedure itself.

*I don’t have a car and the nearest abortion clinic is six hours away. I just began my new job and I couldn’t afford to pay someone to drive me or the $500 fee.*

### 3.2. Insufficient Funds to Select Desired Aspects of Abortion Services

In a related theme, while some were able to raise sufficient funds for the abortion procedure itself, they were unable to afford their desired abortion modality or certain aspects of service delivery such as sedation.

*Tomorrow I go to the clinic to take my first pill for medical abortion. As of Saturday when I take the second set of pills, I will be 10 weeks along. I am very nervous about having the procedure this late, but the surgical option was not financially viable for me. I can hardly afford the medical termination.*

Another person had successfully obtained a medication abortion but could not afford the recommended after-procedure ultrasound:

*It’s been three weeks since I had the abortion, all pregnancy symptoms are gone but I haven’t had money for an ultrasound so I took a urine pregnancy test and this test has a very very faint second line. Could it be because my HGC is dropping low? Is it normal?*

Finally, one Reddit user not only had to forgo sedation, but they were forced to wait longer than desired for their abortion, which was a delay with an attendant emotional toll:

*I don’t qualify for financial assistance at (the clinic). I’ll have to postpone another week—I have a second appointment the following week through my insurance. The surgical (procedure) will cost me $400 and does not include sedation of any kind (I would not be able to afford it). But, worse than the cost and lack of sedation is the fact that I’ll have to endure this pregnancy for an entire week longer…and I’m literally at my breaking point.*

### 3.3. Insurance and Administrative Hurdles Create Unique Barriers

In a third theme, as the quotation immediately above suggests, even those with coverage from private or public insurance reported challenges in navigating payment procedures. The logistical hurdles of paying for an abortion with insurance in some cases could be as cumbersome as self-funding. These challenges affected both the ability to obtain desired abortions and the ways in which people were able to do so, including the timing of the procedure. Such payer-related obstacles included bureaucratic red tape, potential violations of privacy, and restrictions on covering abortion procedures altogether.

For example, one poster had private insurance that failed to cover abortion. Thus, they were hustling to sign up for other coverage in time—which likely prevented them from receiving their abortion in a timely way if at all.

*My appointment is tomorrow, and I found out Friday afternoon that my insurance won’t cover the abortion (pills). I read somewhere that I could apply for a temporary Medical-Cal (sic) but I can’t find much info on how to get it and how quickly I can receive it. As a pregnant woman, will I automatically qualify for it? (Authors’ note: California Medicaid is called MediCal.)*

The following Reddit user was able to get on Medicaid due to their pregnancy but was unsure whether their abortion would be or could be covered. Lack of coverage would have left this person to come up with funds that may not have been available. This person also worried about potential payer backlash:

*I’m going to have the aspiration procedure done on Saturday morning. I had Medicaid approved here in Florida because of pregnancy. Now I’m confused. I previously posted that I’m having to terminate due to my baby having anencephaly.*

*Has anyone been through this? Do you call them and tell them what happened? How long after do they cancel your Medicaid? And if you ever get pregnant again are you blacklisted or something? I’m just scared and nervous and want to make sure I can go to my follow-up appointment after the procedure.*

As with all other themes, insurance-related challenges could also be exacerbated by abortion stigma, which frequently discouraged people from seeking financial support from family members or friends.

*I do have health insurance. It’s just that I’m under my parents’ plan and there is absolutely no way I can let them know about this. If anyone could help me it would be greatly appreciated. I’m terrified. My boyfriend and I are living paycheck to paycheck, so I can’t afford anything over $500. I don’t know what to do.*

### 3.4. Relying on Self-Managed Abortion Options Due to Cost Barriers

In a fourth and final theme, people reported how the inability to afford in-clinic abortion services led them to self-managed, outside-the-system abortion venues. While self-managed abortion can be safe, and while some abortion seekers may prefer it to in-clinic services, the Reddit posts we analyzed described people who might have preferred in-clinic procedures if payment was not a barrier. For example, several reported using Aid Access, which is a non-profit organization based in the United Kingdom that provides access to medication abortion by mail to the U.S. and many other countries. While Aid Access is not free, the cost (usually between $100 and $150) is displayed clearly online, paid upfront, and logistically straightforward.

*I used Aid Access to terminate my pregnancy because I couldn’t afford $500+ for the abortion counseling appointment and medical abortion. I didn’t qualify for aid because my finances look good on paper. But they don’t take into account your rent, health insurance, car payment, student loans, etc.*

Another Reddit poster shared a similar example of obtaining abortion pills by mail:

*I decided to hide (my pregnancy) from my parents and sister–basically, everyone except my boyfriend. Because I had only him, and he didn’t have a job at this time (rare for him) or a car, I knew my options were limited. I had enough savings to go to Planned Parenthood, but it would have depleted my bank account, and we probably wouldn’t be able to get to appointments. So I took the cheaper and easier way, and I ordered mifepristone and misoprostol from overseas.*

This person estimated that they were already 10 weeks pregnant by the time they received the medication, which was “cutting it close” (their words) to the gestational limit for this abortion modality. They had waited for 23 days to receive their Aid Access package, reporting that the pills were “inbound into customs” for more than two weeks. Medication abortions after 10 weeks gestation are associated with heavier cramps and bleeding and greater risk of complications than with earlier gestations.

More concerningly, some Reddit users with few financial resources were considering or inquiring about self-managed abortion techniques that are unsupported by clinical research and that may be both ineffective and harmful.

*I’m not financially fit, mentally ready... but I can’t afford abortion pills. And the closest Planned Parenthood is over 4 hours away... I’m scared about this whole thing. Also someone told me they have animal misoprostol that might work but I don’t think it would?*

Similarly:

*I’ve been using birth control however I didn’t realize one of my medications may make it ineffective. My insurance doesn’t cover abortions unless a physician says I was raped and my bank account is joint with my husband who I’m pretty sure would try to talk me out of aborting. Could I use diclofenac (voltaren) (a medication used to relieve pain and swelling from arthritis)? Someone told me it’s basically misoprostol.*

## 4. Discussion

In this study of Reddit posters, we found multiple financial barriers faced by people seeking abortions. These barriers led to delays in service as well as inquiry into self-managed abortions. While resonating with prior literature [9,15,32], this cumulative analysis captured anonymous sentiments posted by abortion-seekers online, in real time, versus during retrospective data collection. Moreover, we documented cost-related challenges before individuals ever entered an abortion clinic—if they were able to do so at all.

Some posters described self-managed techniques versus clinical venues for abortion care. While receiving and using medication abortion by mail can be safe, effective, and affordable [33,34], it is not legally sanctioned in the U.S. While some people seeking abortion may prefer mail-based services to abortion clinics even if they can access and afford both, Reddit posts documented that the costs and logistics of in-clinic abortions may back people into illegal services such as Aid Access. We mention the illegality of self-managed abortion through Aid Access not to cast pallor on what is a safe abortion modality, but to highlight that people using these methods may be at risk for criminal or legal retribution. Moreover, some posters were looking into supposed abortifacients such as pain medication or veterinary products that are not clinically proven and potentially dangerous. These posters tied their consideration of these self-managed options to the high cost of, and poor access to, in-clinic abortion services. In this way, our study found that people implicated cost-related challenges when they could not terminate their pregnancies when they wished to or how they wished to, in some cases leading to consideration or use of illegal or potentially dangerous self-managed abortion modalities.

While this analysis focused primarily on cost hurdles, it is important to consider these hurdles within the context of other obstructions to desired pregnancy termination [1,35]. One major contextual factor is the intersection of structural disadvantage with abortion. People most likely to experience unwanted pregnancy and most in need of financial assistance for abortion services often face multiple structural hardships, including poverty, racism [5], homelessness [32], mental illness, and substance use [36]. Disruptive life events such as job loss and housing evictions also exacerbate the ability to secure funds and obtain abortion services quickly [37]. In other words, cost barriers are hardest for the most socially and structurally disadvantaged and people in our country, placing abortion out of reach for some and instead leading to live births.

In another important contextual factor, the landscape of state-level abortion restrictions *other* than state Medicaid restrictions work synergistically to exacerbate cost challenges. In many states, patients must make multiple trips to a facility due to mandatory waiting periods, which are required of virtually no other outpatient healthcare procedure save for tubal ligation (but not vasectomy). People living significant distances from a clinic, those with children, and those working in low-wage jobs are especially encumbered by such waiting periods. Non-evidence-based laws mandate heavier regulation of abortion clinics than other outpatient care settings, forcing some clinics to close their doors under the regulatory and financial encumbrances involved (e.g., widening hallways) [38]. When combined with the cost-related barriers outlined here, these restrictions cumulatively stymie reproductive autonomy by putting pregnancy termination services out of reach for many.

### 4.1. Public Health Implications: The Potential Impact of, and Building Momentum for, Overturning Hyde

Given this daunting landscape, no one solution will guarantee abortion access for all. However, cost-related policy change is critical [1]. Repealing the Hyde Amendment and other payer restrictions on abortion would significantly help lower-income individuals obtain desired abortion services [39]. Hyde and state-level prohibitions on public funds for abortion inordinately harms people who rely on public insurance for healthcare coverage, creating a separate system of care that privileges those who can afford out-of-pocket costs or who live in states that do allow state Medicaid funding for abortion. Those who cannot afford out-of-pocket costs often obtain abortions later in gestation or forgo abortion altogether [7]. Furthermore, a disproportionate amount of the estimated seven million people impacted by the Hyde Amendment are people of color, further entrenching reproductive health inequities [40].

Politicians almost universally express their commitment to retaining or overturning Roe v. Wade. However, the Hyde Amendment has long been absent from mainstream political platforms. Hilary Clinton was the first presidential candidate to publicly argue for Hyde’s repeal. Current president Joe Biden historically expressed pro-Hyde sentiments but changed this opinion during his latest campaign, saying “I can no longer support an amendment that makes the right (to healthcare) dependent on someone’s ZIP code [41].” Progressive politicians and reproductive health research and policy organizations have made increasing calls for the removal of payer restrictions on abortion [3,6]. In July 2020, Representative Ayanna Pressley of Massachusetts introduced an amendment to repeal Hyde from the HHS funding bill [42]. In December of 2020, the House Appropriations Labor, Health, and Human Services Subcommittee held a hearing to discuss the effects of the Hyde Amendment, specifically as it affects gender rights and economic security [43]. However, staunch opposition to the Hyde Amendment remains intact [44,45]. In January 2021, a group of 200 House Republican lawmakers signed a letter vowing not to support any funding bill that includes language permitting the use of federal funding for abortion [45]. In 2021, the President’s budget and subsequent House spending bills did not include the Hyde Amendment. While not yet repealed, Hyde had not been formally reconsidered in this way since its initial passage in 1976. However, even if the amendment is removed from the federal budget, significant changes in the Medicaid program will be required before Federal Medicaid coverage extends to abortion [46,47,48].

### 4.2. Limitations

Although Reddit provides both copious and gratis data, the information posted can be thin—sometimes just a sentence or two, leaving us with less complete information than we would have obtained in in-depth interviews. Second, the comments in our dataset came from a convenience sample of self-selected individuals who might have had more reason than the average abortion seeker to focus on cost-related barriers; Reddit posters may be meaningfully different than the larger universe of people who desire abortion services. Third, by scraping usernames to protect people’s identities, we risk a small chance that some separate posts may have come from the same individual. Fourth, we were unable to confirm if people encountering cost challenges were eventually able to procure their abortion, which is a phenomenon that left us open to conjecture versus confirmation. However, the aim of this study was not to determine if people can navigate financial barriers, but to more fully understand the dimensions of those barriers. Reddit data also enabled us to collect information from people who may never have participated in a qualitative research study through more traditional means. Our data demonstrate that many people seeking abortion services and advice turn to online sources such as Reddit, which are sources currently underexamined by researchers and health professionals alike.

## 5. Conclusions

Cost-related prohibitions are likely the largest obstacle to abortion access, especially for individuals who face the greatest social inequities. Even those who have insurance coverage face obstacles, which can delay if not avert procedures [4]. Findings from this analysis of anonymous Reddit posters suggest that cost barriers led some to consider illegal and, in some cases, harmful self-managed abortion techniques. We encourage public health scholars, practitioners, and leaders to emphasize the ill effects of abortion cost restrictions—in particular, how the Hyde Amendment impedes low-income individuals’ ability to exercise their right to abortion care and reproductive autonomy. Despite the bleak context of many of these posts around the costs of abortion services, our group’s ongoing work with these data examine the posters’ community and support for each other on Reddit, which is an online forum that allows people an anonymous space to discuss an otherwise stigmatized topic. Future abortion research and advocacy would benefit from further engagement with online sources [49], especially social media, where people seeking abortion turn for support and information.
